# Long-acting antimuscarinic therapy in patients with chronic obstructive pulmonary disease receiving beta-blockers

**DOI:** 10.1186/s12931-021-01861-2

**Published:** 2021-10-22

**Authors:** Kenneth R. Chapman, Robert A. Wise, Benjamin M. Scirica, Deepak L. Bhatt, Sami Z. Daoud, Dan Lythgoe, Esther Garcia Gil

**Affiliations:** 1 Asthma and Airway Centre, University of Toronto, Toronto, ON Canada; 2 Pulmonary Function Laboratory, Johns Hopkins University School of Medicine, Baltimore, MD USA; 3 Division of Cardiovascular Medicine, Brigham and Women’s Hospital, Harvard Medical School, Boston, MA USA; 4 Late-Stage Respiratory and Immunology, BioPharmaceuticals R&D, AstraZeneca, Gaithersburg, MD USA; 5 Statistics, Phastar, Chiswick, London, UK; 6 Formerly of AstraZeneca, Barcelona, Spain; 7 Asthma and Airway Centre, University Health Network, 7th Floor East Wing, 399 Bathurst St, Toronto, ON M5T 2S8 Canada

**Keywords:** Aclidinium, Beta-adrenergic antagonists, Cardiac risk, Exacerbations

## Abstract

**Background:**

Beta-blocker therapies for cardiovascular comorbidities are often withheld in patients with chronic obstructive pulmonary disease (COPD) due to potential adverse effects on airway obstruction. We carried out a post hoc analysis to determine the efficacy and safety of aclidinium in patients with moderate-to-very severe COPD and increased cardiovascular risk receiving beta-blockers at baseline versus non-users.

**Methods:**

ASCENT-COPD was a Phase 4, multicenter, double-blind, randomized, placebo-controlled, parallel-group study. Patients were randomized 1:1 to aclidinium or placebo twice-daily for up to 3 years. Outcomes included risk of (time to first) major adverse cardiovascular events (MACE), all-cause mortality, and lung function over 3 years, and exacerbations over 1 year.

**Results:**

Of 3589 patients, 1269 (35.4%) used beta-blockers and 2320 (64.6%) were non-users at baseline. Aclidinium did not statistically increase the risk of MACE (beta-blocker user: hazard ratio 1.01 [95% CI 0.62–1.64]; non-user: 0.80 [0.51–1.24]; interaction *P* = 0.48) or all-cause mortality (beta-blocker user: 1.13 [0.78–1.64]; non-user: 0.89 [0.62–1.26]; interaction *P* = 0.35), in patients using beta-blockers. Aclidinium reduced annualized rate of moderate-to-severe COPD exacerbation (beta-blocker user: rate ratio 0.75 [95% CI 0.60–0.94, *P* = 0.013]; non-user: 0.79 [0.67–0.93, *P* = 0.005]), delayed time to first exacerbation, and improved lung function versus placebo. There was greater trough FEV_1_ benefit in beta-blocker users versus non-users (least squares mean difference at 52 weeks: 111 mL [95% CI 74 mL–147 mL] versus 69 mL [42 mL–97 mL]; interaction *P* = 0.041).

**Conclusions:**

This post hoc analysis supports long-acting anti-muscarinic use with concomitant beta-blockers in patients with moderate-to-very severe COPD and cardiovascular comorbidity.

*Trial registration:* ClinicalTrials.gov, NCT01966107, Registered 16 October 2013, https://clinicaltrials.gov/ct2/show/NCT01966107.

## Background

Chronic obstructive pulmonary disease (COPD) is a leading cause of morbidity and mortality worldwide and is characterized by airflow limitation, chronic inflammation, and lung tissue damage (emphysema) [1]. Many patients with COPD have comorbidities that can affect their prognosis [2–4]. Cardiovascular (CV) comorbidities are particularly prevalent in patients with COPD [5–8], in part due to common risk factors, such as smoking, age, and environmental pollutants, shared genetic susceptibility, and systemic inflammation [9, 10].

Beta-blockers are commonly used to manage a range of CV conditions and act by preventing the stimulation of beta-adrenergic receptors, thereby antagonizing endogenous catecholamine responses in the heart [11]. Inhaled short- and long-acting β_2_-agonists (LABAs) are a key therapy for COPD, particularly for patients experiencing exacerbations [1]. Despite the availability of cardio-selective beta-blockers, they are often withheld in patients with COPD over concerns of worsening airway obstruction through β_2_-receptor antagonism [12, 13].

Long-acting muscarinic antagonists (LAMAs), such as tiotropium and aclidinium, are commonly recommended and prescribed as maintenance bronchodilator therapies for COPD and achieve bronchodilation via blocking airway M_3_ muscarinic acetylcholine receptors [1]. Due to their different mechanisms of action, LAMAs and LABAs can produce synergistic effects when combined [14]; however, there has been some uncertainty as to whether treatment with a LAMA is associated with an increased risk of CV events [15–19]. This may in part be because LAMAs also show some affinity for the M_1_ and M_2_ receptors, which are located outside the respiratory tract [20]. Indeed, blockade of cardiac M_2_ receptors has been associated with tachycardia [21]. However, it is worth noting that aclidinium has been found to have a reduced affinity for M_2_ receptors compared with other LAMAs [22]. In addition, although β-adrenoreceptor antagonists (non-selective beta-blockers) are indicated for the treatment of heart failure and post-myocardial infarction [23], they may result in bronchoconstriction and thus there is frequently a reticence to use them in patients with COPD [24].

The ASCENT-COPD trial was a Phase 4, multicenter, double-blind, randomized, placebo-controlled, parallel-group non-inferiority study assessing the effects of aclidinium on CV safety and COPD exacerbation risk in patients with moderate-to-very severe COPD and an elevated risk of CV events [25]. Aclidinium bromide 400 µg twice daily did not increase the risk of major adverse cardiovascular events (MACE) versus placebo over 3 years, and reduced the rate of COPD exacerbations versus placebo over the first year. This post hoc analysis aimed to evaluate the effect of aclidinium on the risk of (time to first) MACE, all-cause mortality, COPD exacerbations, and lung function in patients with moderate-to-severe COPD and CV risk factors who were receiving beta-blockers at baseline versus those who were not.

## Methods

### Study design

The methodology of ASCENT-COPD has been previously reported in detail [26]. In brief, ASCENT-COPD (NCT01966107) was a Phase 4, multicenter, double-blind, randomized, placebo-controlled, parallel-group study conducted at 522 sites in the USA and Canada [25]. The study comprised a 2-week washout period followed by a double-blind treatment phase, during which patients were randomized with equal allocation to receive aclidinium 400 μg or placebo twice daily, administered via a multidose dry powder inhaler (Genuair/Pressair; AstraZeneca) for up to 3 years. The trial protocol and informed consent procedures were approved by the institutional review board with controlling authority at each study site. All patients provided written informed consent prior to the conduct of any study-specific procedures. The study was performed in accordance with the Declaration of Helsinki and Good Clinical Practice Guidelines.

In this post hoc analysis, patients were analyzed according to whether they were receiving beta-blockers or not at baseline.

### Study population

The study population for the ASCENT-COPD study has been described previously [25]. Eligible patients were aged ≥ 40 years with stable moderate-to-very severe COPD (forced expiratory volume in 1 s/forced vital capacity [FEV_1_/FVC] < 0.70, and FEV_1_ < 80% predicted) and ≥ 10 pack-years of smoking history, plus ≥ 1 of the following CV risk factors: cerebrovascular disease (eg, stroke or transient ischemic attack, carotid stenosis); coronary artery disease (eg, angina, myocardial infarction, angioplasty/stent/bypass); or peripheral vascular disease (stent/bypass or claudication). Alternatively, patients could have ≥ 2 of the following atherothrombotic risk factors: age ≥ 65 years for men, ≥ 70 years for women; waist circumference ≥ 40 inches for men, ≥ 38 inches for women; estimated glomerular filtration rate < 60 mL/min and microalbuminuria (defined as ≥ 30–300 μg albumin/mg creatinine on a spot urine test or 30–300 mg albumin on a 24-h urine test); dyslipidemia; diabetes; or hypertension.

Key exclusion criteria included: receiving triple therapy (inhaled corticosteroid [ICS]/LABA/LAMA); a respiratory infection or COPD exacerbation at screening or within 4 weeks prior to screening; unstable or life-threatening COPD or CV disease; comorbid lung disease other than COPD; planned lung transplant or lung volume reduction surgery; or malignancy requiring intervention within 5 years prior to screening.

Patients were initially required to have had one or more treated COPD exacerbation in the year prior to screening; however, this requirement was removed after approximately half of the patients were enrolled to increase accrual and allow for a broader patient population. At that time, the upper limit of FEV_1_ was also increased from 70 to 80% predicted.

### Outcomes

Safety outcomes included time to first MACE (on-study analyses), as adjudicated by a clinical endpoint adjudication committee, and all-cause mortality over 3 years [25]. Efficacy outcomes included the annualized rate of moderate-to-severe COPD exacerbations and time to first moderate-to-severe COPD exacerbation during the first year, and change from baseline in morning pre-dose (trough) FEV_1_ over 3 years (on-treatment analyses). COPD exacerbations were defined as increased COPD symptoms lasting two or more days that required treatment with antibiotics and/or systemic corticosteroids, or led to hospitalization or death [25].

### Statistical analyses

All statistical models included the main effect of randomized treatment and the interaction between treatment group and beta-blocker use at baseline to enable a comparison of treatment effects between subgroups. Time to first MACE, all-cause mortality, and time to first moderate-to-severe COPD exacerbation were analyzed using Cox regression models adjusted for baseline CV severity and smoking status. Moderate-to-severe COPD exacerbation rates were analyzed using a negative binomial regression model adjusted for baseline ICS use, baseline COPD severity, prior 1-year exacerbation history, and smoking status. Spirometry outcomes were analyzed using linear mixed models adjusted for pre- and post-bronchodilator FEV_1_ at screening, baseline FEV_1_, smoking status, baseline ICS use, visit, treatment-by-visit, and treatment-by-beta-blocker-by-visit interactions, and assuming an unstructured covariance matrix for the visits.

The full analysis set included all patients randomized to treatment who received ≥ 1 dose of study treatment irrespective of treatment discontinuation. On-treatment analyses included events that occurred while patients were exposed to study treatment, whereas on-study analyses included all events that occurred while patients were in the study, irrespective of treatment exposure [26].

All reported outputs were produced using SAS version 9.3 (SAS Institute Inc) in a secure, validated environment. The significance threshold was *P* < 0.05 for comparisons between aclidinium and placebo; *P* < 0.10 for the interaction between treatment group and beta-blocker use at baseline was considered statistically significant for evidence of a different treatment effect across beta-blocker groups.

### Role of the funding source

Forest Laboratories were involved in the study design, collection, and analysis of data. AstraZeneca was involved in data collection and interpretation, and the development and review of this manuscript. The decision to submit the manuscript for publication was made by the authors.

## Results

### Baseline demographics and characteristics

Of the 3589 patients included in this analysis, 1269 (35.4%) were beta-blocker users and 2320 (64.6%) were non-users at baseline. Baseline characteristics were generally similar between groups (Table 1); however, a greater proportion of beta-blocker users were male (64.4% vs 55.5%) and had one or more prior CV event (67.9% vs 36.7%) than non-users. Baseline COPD characteristics were similar between beta-blocker users and non-users in terms of post-bronchodilator FEV_1_ (mean % predicted, standard deviation [SD] 47.2 [14.2] vs 48.0 [15.4]), pre-bronchodilator FEV_1_ (mean [SD] 1215 mL [486] vs 1213 mL [492]), the percentage of patients experiencing exacerbations (60.5% vs 59.8%), and the exacerbation rate in the prior year (mean [SD] 0.8 [0.9] vs 0.8 [1.0]). Of note, concomitant use of LABA therapies, which were started after the first study dose, was similar between beta-blocker users compared with non-users (eg, LABA/ICS: 10.5% vs 9.6%; LABA monotherapy: 0.6% vs 0.6%, respectively). Cardio-selective beta-blocker use was more common than non-selective beta-blocker use (69.9% vs 30.7% of patients respectively); of note, up to 11 patients took multiple beta-blockers (Table 2). Metoprolol was the most common beta-blocker used (49.4%).

**Table 1:** Patient demographics and characteristics (full analysis set)

	Beta-blocker users (*N* = 1269)	Non-users (*N* = 2320)
*Patients*		
Age, years, mean (SD)	68.1 (7.9)	66.6 (8.6)
Male, %	64.4	55.5
White, %	90.6	90.7
BMI, kg/m^2^, mean (SD)	30.4 (6.7)	29.1 (6.8)
Obese (≥ 30 kg/m^2^), n (%)	597 (47.0)	913 (39.4)
Current smoker, %	39.8	45.6
Prior CV events, %	67.9	36.7
Pre-BD FEV_1_ (mL), mean (SD)	1215 (486)	1213 (492)
Post-BD FEV_1_% predicted, mean (SD)	47.2 (14.2)	48.0 (15.4)
COPD severity, ^a^ %		
Moderate	42.0	46.2
Severe	44.5	38.4
Very severe	11.9	13.5
COPD exacerbations in previous year, %	60.5	59.8
≥ 2 prior COPD exacerbations, %	16.6	15.1
Exacerbation rate in previous year, mean (SD)	0.8 (0.9)	0.8 (1.0)
Prior and concomitant COPD medication, ^b^ n (%)		
LABA + ICS	706 (55.6)	1310 (56.5)
SABA	638 (50.3)	1227 (52.9)
ICS	125 (9.9)	200 (8.6)
LABA	77 (6.1)	150 (6.5)
Systemic corticoids	31 (2.4)	86 (3.7)
SAMA	5 (0.4)	11 (0.5)
SABA + SAMA	4 (0.3)	12 (0.5)
LAMA	2 (0.2)	7 (0.3)
Monoclonal antibody	1 (0.1)	0 (0.0)
LABA + LAMA	1 (0.1)	1 (< 0.1)

Full analysis set (*N* = 3589); included all patients randomized to treatment who received ≥ 1 dose of study drug

*BD* bronchodilator, *BMI* body max index, *COPD* chronic obstructive pulmonary disease, *CV* cardiovascular, *FEV*_*1*_ forced expiratory volume in 1 s, *GOLD* Global Initiative for Chronic Obstructive Lung Disease, *ICS* inhaled corticosteroid, *LABA* long-acting β_2_-agonist, *LAMA* long-acting muscarinic antagonist, *N* number of subjects in treatment group, *n* number of subjects in category or analysis, *SABA* short-acting β_2_-agonist, *SAMA* short-acting muscarinic antagonist, *SD* standard deviation

^a^Based on GOLD airflow obstruction grade: moderate (50% ≤ FEV_1_ < 80% predicted); severe (30% ≤ FEV_1_ < 50% predicted); very severe (FEV_1_ < 30% predicted)[1]

^b^Medications started prior to randomization and continued after first dose of study drug

**Table 2:** Beta-blocker medication (full analysis set)

	Total patients (*N* = 3589)
*Medication*	
Patients who took any beta-blocker medications, n (%)	1269 (35.4)
Selective beta-blocking agents	889 (24.7)
Metoprolol^a^	627 (17.4)
Atenolol^a^	171 (4.8)
Bisoprolol^a^	56 (1.6)
Nebivolol^a^	34 (0.9)
Acebutolol	1 (< 0.1)
Non-selective beta-blocking agents	391 (10.9)
Carvedilol	314 (8.7)
Propranolol^a^	39 (1.1)
Sotalol^a^	24 (0.7)
Labetalol	9 (0.3)
Nadolol	4 (0.1)
Pindolol with hydrochlorothiazide	1 (< 0.1)

Full analysis set (*N* = 3589); included all patients randomized to treatment who received ≥ 1 dose of study drug

Beta-blocker medication was taken before randomization and continued after the first study dose. Of note, up to 11 patients took multiple beta-blockers across this period

*N* number of subjects in treatment group, *n* number of subjects in category

^a^Includes different salt conjugates and diuretic combinations

Pre-bronchodilator FEV_1_ was similar for beta-blocker users and non-users (1255 mL and 1247 mL, respectively, in the placebo arm).

### Safety

**Fig. 1:**
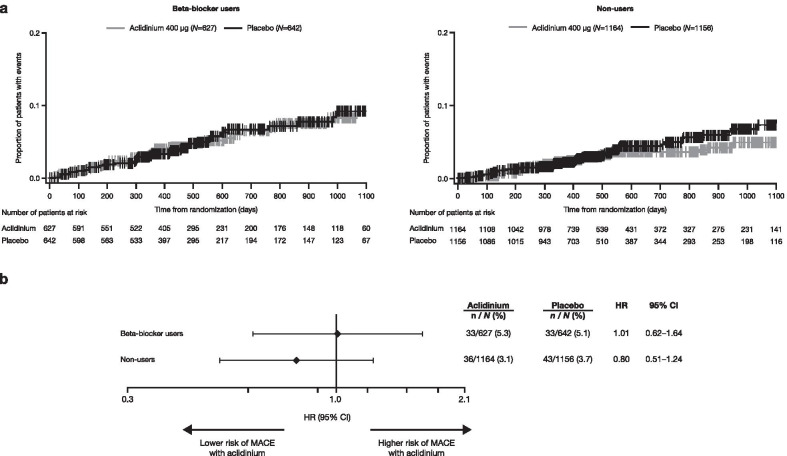
Kaplan–Meier plot (**a**) and Cox regression analysis (**b**) of time to first adjudicated MACE. Cox proportional hazards model with baseline CV risk group, smoking status, treatment group and baseline beta-blocker use as factors. Interaction *P* = 0.48. *CI* confidence interval, *CV* cardiovascular, *HR* hazard ratio, *MACE* major adverse cardiovascular event, *N* total number of patients, *n* total number of patients experiencing event

Numerically, a higher proportion of beta-blocker users had MACE compared with non-users (5.2% vs 3.4%, respectively); however, the effect of aclidinium treatment on MACE did not differ between beta-blocker users and non-users. Neither group were at increased risk of MACE when treated with aclidinium versus placebo (hazard ratio [HR] 1.01 [95% CI 0.62–1.64] and 0.80 [0.51–1.24], respectively; interaction *P* = 0.48; Fig. 1).

**Fig. 2:**
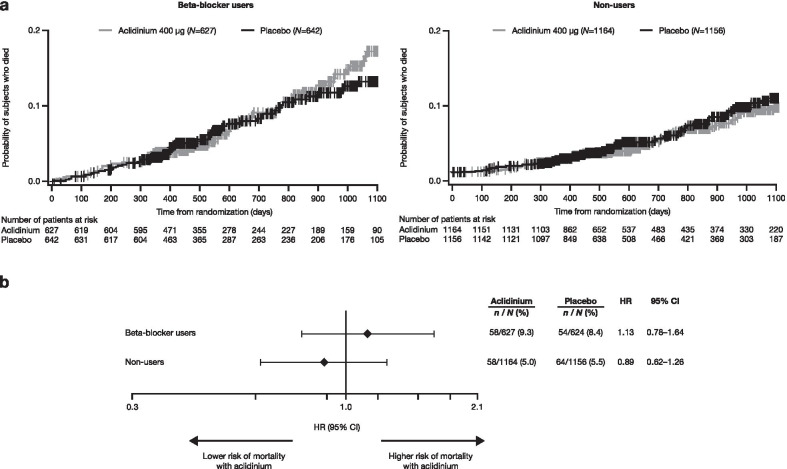
Kaplan–Meier plot (**a**) and Cox regression analysis (**b**) of all-cause mortality. Cox proportional hazards model with baseline CV risk group, smoking status, treatment group and baseline beta-blocker use as factors. Interaction *P* = 0.35. *CI* confidence interval, *CV* cardiovascular, *HR* hazard ratio, *N* total number of patients, *n* total number of patients experiencing event

Similarly, there was a numerically higher proportion of deaths for beta-blocker users compared with non-users (8.8% vs 5.3%, respectively); however, there was no increased risk of death when treated with aclidinium versus placebo, regardless of beta-blocker use (Fig. 2). The HR for all-cause mortality was 1.13 (95% CI 0.78–1.64) for beta-blocker users, and 0.89 (95% CI 0.62–1.26) for non-users (interaction *P* = 0.35).

### Efficacy

**Fig. 3:**
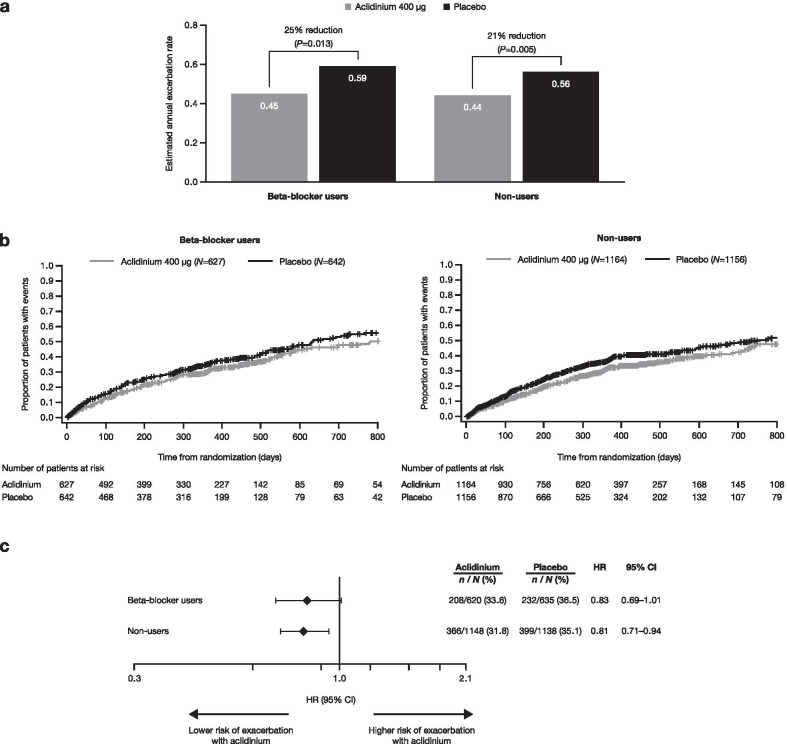
Moderate-to-severe COPD exacerbation rate during first year of treatment (**a**), Kaplan–Meier plot (**b**) and Cox regression analysis (**c**) of time to first moderate-to-severe COPD exacerbation. Cox proportional hazards model with treatment group, baseline ICS use, baseline COPD severity, smoking status, 1-year exacerbation history and beta-blocker use at baseline as factors. Moderate-to-severe COPD exacerbation rate during first year of treatment interaction *P* = 0.75; Cox regression analysis interaction *P* = 0.83. *CI* confidence interval, *COPD* chronic obstructive pulmonary disease, *HR* hazard ratio, *ICS* inhaled corticosteroids, *N* total number of patients, *n* total number of patients experiencing event

Compared with placebo, aclidinium reduced the annualized rate of moderate-to-severe COPD exacerbations in both beta-blocker users and non-users (Fig. 3a), with no differential treatment effect (rate ratio 0.75 [95% CI 0.60–0.94, *P* = 0.013] and 0.79 [0.67–0.93, *P* = 0.005], respectively; interaction *P* = 0.75). In addition, the time to first moderate-to-severe COPD exacerbation was delayed for aclidinium versus placebo, regardless of beta-blocker use (Fig. 3b, c). The HR for beta-blocker users was 0.83 (95% CI 0.69–1.01, *P* = 0.058), and 0.81 for non-users (95% CI 0.71–0.94, *P* = 0.004; interaction *P* = 0.83).

**Fig. 4:**
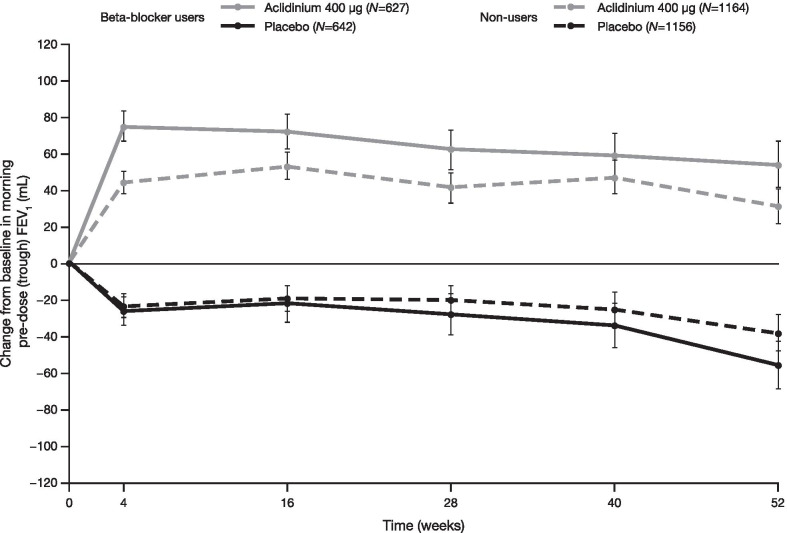
Change from baseline in morning pre-dose (trough) FEV_1_ in beta-blocker users and non-users. Baseline was the average of two pre-dose values prior to administration of first dose, or one value if only one was available, or pre-dose bronchodilator value at screening if both were missing. Change in baseline FEV_1_ (LS mean ± standard error) analysis was based on a mixed model for repeated measures with pre- and post-bronchodilator values at screening, baseline FEV_1_ as covariates, and treatment group, smoking status, baseline ICS use, baseline beta-blocker use and visits as fixed effects. On-treatment analysis during the first year included patients who completed 1 year or were on-treatment when the study dropped off. *FEV*_*1*_ forced expiratory volume in 1 s, *ICS* inhaled corticosteroids, *LS* least squares

Improvement from baseline in trough FEV_1_ was significantly greater with aclidinium versus placebo at all visits (Fig. 4); beta-blocker users derived a significantly greater benefit from aclidinium treatment than non-users (least squares [LS] mean difference at 52 weeks: 111 mL [95% CI 74 mL–147 mL] and 69 mL [42 mL–97 mL], respectively, both *P* < 0.001; interaction *P* = 0.041). A greater benefit was also observed for FEV_1_ expressed as a percentage of predicted normal values in beta-blocker users (LS mean difference at 52 weeks: 4.3 [95% CI 2.93–5.62, *P* < 0.001]) compared with non-users (LS mean difference at 52 weeks: 2.7 [95% CI 1.73–3.75, *P* < 0.001; interaction *P* = 0.049]).

## Discussion

In this post hoc analysis of the ASCENT-COPD study, risk of MACE and all-cause mortality were found to be similar for aclidinium versus placebo, regardless of concomitant beta-blocker use. Aclidinium delayed the time to first moderate-to-severe COPD exacerbation and reduced the annualized exacerbation rate to a similar extent in beta-blocker users and non-users. In addition, aclidinium improved lung function versus placebo, in beta-blocker users and non-users, with significantly greater treatment benefit observed in beta-blocker users; a finding of clinical relevance that has not been reported previously. It is possible that the increase in absolute FEV_1_ could be attributed to a greater proportion of the male sex in the beta-blocker user group; however, similar results were observed in the percent predicted values, which were adjusted for sex and height.

These results were similar to those from a recent post hoc analysis of the TONADO study, which found that the efficacy and safety of tiotropium/olodaterol was not affected by concomitant beta-blocker use in patients with moderate-to-very severe COPD [27]. In addition, a further study demonstrated a lack of association between beta-blocker use and increased exacerbations following a myocardial infarction [28].

CV and cerebrovascular comorbidities are common in patients with COPD [1, 8, 29]. Only one third of the study population, chosen for their history of vascular disease, were beta-blocker users, consistent with reports that these agents are often withheld in patients with COPD over concerns of worsening airway obstruction [12, 13]. There was no difference in baseline lung function between patients with moderate-to-very severe COPD according to beta-blocker use; however, greater improvements in lung function were observed following aclidinium inhalation in patients using background beta-blocker therapy, compared with those who were not. Such a result could be explained by sub-optimal β2-agonist bronchodilator effect in beta-blocker users and argues that in patients with COPD concurrently treated with bronchodilators, an anticholinergic bronchodilator should be part of the treatment regimen. In an exaggerated form, such a phenomenon has been described in asthma, where bronchoconstriction treated by the inadvertent administration of a beta-blocker responds better to anticholinergic therapy than β_2_-agonist therapy [30, 31].

Approximately 70% of the beta-blockers used during this study were cardio-selective. Further investigation into any potential differences, particularly in FEV_1_, for cardio-selective versus non-selective beta-blockers is warranted; however, this was not performed in this study due to the low number of patients taking non-selective beta-blockers.

This post hoc analysis underscores the findings of the primary study in that the use of beta-blockers identified a subpopulation of patients with a higher incidence of CV risk factors. Patients using beta-blockers had higher mortality risk compared with patients not using these agents, likely reflective of an increased incidence of prior vascular events, occurrence of MACE during the study, prescription bias and perhaps increased percentage of male sex. Nonetheless, even in this higher risk subpopulation, aclidinium did not increase MACE and improved COPD outcomes.

It is our understanding that this is the first study using a LAMA that has actively sought to include patients with COPD with a CV risk factors. Strengths of this study include the design, which had an efficacy outcome nested within it, allowing for risk–benefit comparisons within the same patient population. Some limitations of the current study should be noted. First, a relatively small number of patients were studied beyond one-year post-randomization. Second, the LAMA used in this study is rapidly hydrolyzed with low potential for systemic effect [32], as reflected by low reported rates of dry mouth and urinary retention; findings with aclidinium should therefore be extrapolated cautiously. Finally, this was a post hoc subgroup analysis with no corrections for multiple statistical comparisons, and therefore should be considered hypothesis-generating. Of note, both MACE and all-cause mortality, covariates were identified a priori as being the most important variables in the original ASCENT study; these included treatment group, baseline CV severity, smoking status, and baseline beta-blocker use, along with the interaction between beta-blocker use at baseline and treatment group. In addition, as within subgroup comparisons were randomized, group comparisons were deemed valid. However, to further account for confounding factors, multivariate analyses could be used to explore differences between cardioselective and non-cardioselective beta-blockers.

## Conclusions

In this post hoc analysis of the ASCENT-COPD study of patients with moderate-to-severe COPD and increased CV risk, beta-blocker users and non-users had similar pre-bronchodilator FEV_1_. The safety of aclidinium versus placebo, in terms of risk of (time to first) MACE and all-cause mortality, was not influenced by beta-blocker use. Treatment with aclidinium reduced the rate of COPD exacerbations, delayed the time to first exacerbations, and improved lung function regardless of beta-blocker use, with a greater benefit in FEV_1_ observed in beta-blocker users versus non-users. This analysis supports the use of LAMAs with concomitant beta-blockers in patients with moderate-to-severe COPD and CV comorbidity.

## Data Availability

Data underlying the findings described in this manuscript, including individual deidentified participant data, protocols and clinical trial documents, may be obtained in accordance with AstraZeneca’s data-sharing policy (described at https://astrazenecagrouptrials.pharmacm.com/ST/Submission/Disclosure) through Vivli (https://vivli.org/).

## Abbreviations

BD: Bronchodilator
BMI: Body mass index
CI: Confidence interval
COPD: Chronic obstructive pulmonary disease
CV: Cardiovascular
FEV_1_: Forced expiratory volume in 1 s
FVC: Forced vital capacity
GOLD: Global Initiative for Chronic Obstructive Lung Disease
HR: Hazard ratio
ICS: Inhaled corticosteroid
LABA: Long-acting β_2_-agonists
LAMA: Long-acting muscarinic antagonists
LS: Least squares
MACE: Major adverse cardiovascular events
SABA: Short-acting β_2_-agonist
SAMA: Short-acting muscarinic antagonist
SD: Standard deviation
